# A randomized, placebo-controlled, phase 1 study to evaluate the effects of TAK-063 on ketamine-induced changes in fMRI BOLD signal in healthy subjects

**DOI:** 10.1007/s00213-019-05366-1

**Published:** 2019-11-26

**Authors:** Deborah A. Yurgelun-Todd, Perry F. Renshaw, Paul Goldsmith, Tolga Uz, Thomas A. Macek

**Affiliations:** 1 The Brain Institute, University of Utah, 383 Colorow Drive, Salt Lake City, UT 84108 USA; 2 Takeda Development Center Europe, Ltd., 61 Aldwych, London, WC2B 4AE UK; 3 Takeda Development Center Americas, Inc., One Takeda Parkway, Deerfield, IL 60015 USA

**Keywords:** fMRI, BOLD, Ketamine, TAK-063, PDE-10 inhibitor

## Abstract

**Rationale:**

Phosphodiesterase 10A inhibitor TAK-063 has shown effects that suggest efficacy in schizophrenia treatment.

**Objective:**

This randomized, double-blind, placebo-controlled, incomplete-crossover study investigated effects of single oral administration of TAK-063 on ketamine-induced changes in blood oxygen level-dependent (BOLD) signal in healthy males.

**Methods:**

Healthy men aged 18 to 45 years with normal magnetic resonance imaging (MRI) scans and electroencephalogram measurements at screening were eligible. Each subject was randomized to one of nine treatment schedules: all subjects received placebo and two of three doses of TAK-063 followed by ketamine. The primary endpoint was ketamine-induced brain activity in select regions of the brain during resting state. Secondary endpoints included pharmacokinetic parameters of TAK-063, proportion of subjects with treatment-emergent adverse events (AEs), and percentage of subjects meeting criteria for abnormal safety laboratory tests and vital sign measurements.

**Results:**

The study comprised 27 subjects. Prior to ketamine infusion, TAK-063 exerted region-specific effects on resting state functional MRI (fMRI) BOLD signal. After ketamine administration, TAK-063 reduced the Cohen’s effect size for resting-state fMRI BOLD signal in key brain regions examined, and exerted similar effects on BOLD signal during the working memory task across all doses. TAK-063 was safe and well tolerated.

**Conclusions:**

Our results are consistent with non-clinical studies of ketamine and TAK-063 and clinical studies of ketamine and risperidone. It is unknown whether these data are predictive of potential antipsychotic efficacy, and further analyses are required.

**Electronic supplementary material:**

The online version of this article (10.1007/s00213-019-05366-1) contains supplementary material, which is available to authorized users.

## Introduction

Schizophrenia is a complex disorder consisting of positive symptoms (paranoia, hallucinations, suspiciousness, and unusual thought content), negative symptoms (blunted affect, emotional withdrawal, and poor rapport), and cognitive impairments (attentional and memory deficits, impairments in executive function, and others) (Citrome 2014). Schizophrenia imposes a significant financial and humanistic burden because of factors such as institutionalization and adverse side effects from chronic medication use (Millier et al. 2014). Current antipsychotics have shown efficacy in treating positive symptoms associated with schizophrenia, but have had limited success with treating negative and cognitive symptoms (Citrome 2014). Thus, new treatment options with improved safety and tolerability profiles are needed to address these poorly controlled domains within schizophrenia (Citrome 2014).

Ketamine, an N-methyl-D-aspartate (NMDA) receptor antagonist, is used clinically as a dissociative anesthetic (Yamakura et al. 2000). Ketamine also increases psychotic symptoms in individuals with schizophrenia (Lahti et al. 2001). Sub-anesthetic doses of ketamine induce effects that resemble schizophrenia symptoms in healthy individuals, and have therefore been used as a model for schizophrenia in humans (Abi-Saab et al. 1998; Lahti et al. 2001) (reviewed in Frohlich and Van Horn 2014). Specifically, ketamine impairs working memory and psychomotor function in healthy volunteers (Driesen et al. 2013; Lofwall et al. 2006; Morgan et al. 2004) and induces changes in blood oxygen level-dependent (BOLD) signal as measured by functional magnetic resonance imaging (fMRI) (De Simoni et al. 2013; Driesen et al. 2013). In previous studies, ketamine-induced changes in BOLD signal have been shown to be reversed by risperidone, a dopamine receptor antagonist and atypical antipsychotic (Doyle et al. 2013; Shcherbinin et al. 2015).

TAK-063 is a selective inhibitor of phosphodiesterase 10A (PDE10A), an intracellular enzyme selectively expressed in medium spiny neurons of the striatum (Seeger et al. 2003; Suzuki et al. 2015). In preclinical studies in combination with NMDA antagonists, TAK-063 has been shown to have potential antipsychotic effects in animal models of schizophrenia (Suzuki et al. 2016). For example, TAK-063 reversed MK-801-induced effects on hyperactivity in rats (Suzuki et al. 2016), as well as MK-801 and phencyclidine-induced effects on spatial working memory, attentional set-shifting, and executive function in rodent models of cognitive impairment (Shiraishi et al. 2016). TAK-063 also increased percent BOLD signal change during the resting state in rat caudate putamen and substantia nigra, and reduced ketamine-induced increases in percent BOLD signal change in cortical regions (hippocampus, cingulate [frontal] and retrosplenial [parietal] cortex) in anesthetized rats (Tomimatsu et al. 2016), suggesting that TAK-063 may alleviate physiological features associated with schizophrenia.

In clinical studies, TAK-063 has been shown to be safe and well tolerated (Goldsmith et al. 2017; Tsai et al. 2016). In addition, TAK-063 has shown effects in patients with schizophrenia that are suggestive of potential efficacy in the treatment of this disorder, including increases in electroencephalogram (EEG) gamma power, improvements in mismatch negativity, and some improvements in cognition (Macek et al. 2016a, b).

Although TAK-063 has shown signs of efficacy in preclinical models, its effects in the clinical setting have not been extensively characterized. In a study with a similar design to the one we report here, ketamine was shown to produce a broad increase in BOLD signal; co-administration of risperidone reduced the effect of ketamine on BOLD signal across most regions of interest (ROIs), including the medial prefrontal and cingulate regions and the thalamus (Doyle et al. 2013). Based on the pharmacological properties of TAK-063 and the potential antipsychotic activity observed in preclinical and clinical studies, it was predicted that TAK-063 would have similar effects on the ketamine-induced increase in BOLD signal. We hereby report the effects of TAK-063 on ketamine-induced brain changes in BOLD signal during the resting state and working memory tasks in healthy male subjects.

## Methods

### Study design and subjects

This was a randomized, placebo-controlled, investigator- and subject-blinded, three-period, incomplete crossover, single-center, phase 1 study that was conducted in the USA from June 27, 2013, to August 28, 2014. Data were collected at the University of Utah Neuropsychiatric Institute (Salt Lake City, UT), and the protocol was performed in compliance with Good Clinical Practice regulations and the Declaration of Helsinki. The Institutional Review Board’s written approval of the protocol was received before commencement of this study. Informed consent was obtained from all individual participants included in the study. The study is registered as Effects of TAK-063 on Preventing Ketamine-Induced Brain Activity Changes as Well as Psychotic-Like Symptoms in Healthy Male Adults (Clinical Trials ID: NCT01892189; https://clinicaltrials.gov/ct2/show/NCT01892189).

Healthy adult men aged 18 to 45 were eligible for the study if they had a body mass index (BMI) between 18 and 32 kg/m^2^, as well as normal magnetic resonance imaging (MRI) and EEG measurements at screening. Subjects were excluded if they had received any investigational compound or ketamine within 30 days and had an uncontrolled clinically significant neurologic, metabolic, or psychiatric disorder or other anomaly (including on MRI or EEG), which may have confounded study results. Subjects were also excluded if they had a history of drug or alcohol abuse or dependence, a positive result for illicit drugs or alcohol at screening or check-in, use of nicotine-containing products within 28 days before check-in, or a history of psychiatric disorders such as depression, bipolar disorder, and schizophrenia.

The Statistics Department at Takeda Development Center Americas, Inc., generated a randomization schedule before the start of the study, and this randomization schedule maintained the investigational drug blind. During randomization, subjects were assigned a four-digit randomization sequence that encoded assignment of subjects to the treatment schedule. Subjects were randomized to one of nine treatment schedules and received placebo + ketamine (regimen A) and two of the following three treatment regimens: 3 mg of TAK-063 + ketamine (regimen B), 30 mg of TAK-063 + ketamine (regimen C), and 300 mg of TAK-063 + ketamine (regimen D). This design was updated to change the 300-mg TAK-063 dose to 10 mg in regimen D following the observation of higher than expected rates of dystonia and nausea; the 300-mg dose was not included in the analysis. Treatment sequences are presented in Online Resource Supplementary Table S1. The study consisted of multiple visits with a different treatment protocol on each visit. The visits were standardized and included a screening period (day − 28 to day − 2), check-in (day − 1), a treatment period (days 1), completion of treatment visit (day 2), and a follow-up visit (day 14 ± 3 days after last TAK-063 dose). After each treatment period, subjects stayed overnight at the study site for observation and were discharged on day 2. Each participant completed the study when they completed the follow-up visit after their last treatment2.

**Fig. 1:**
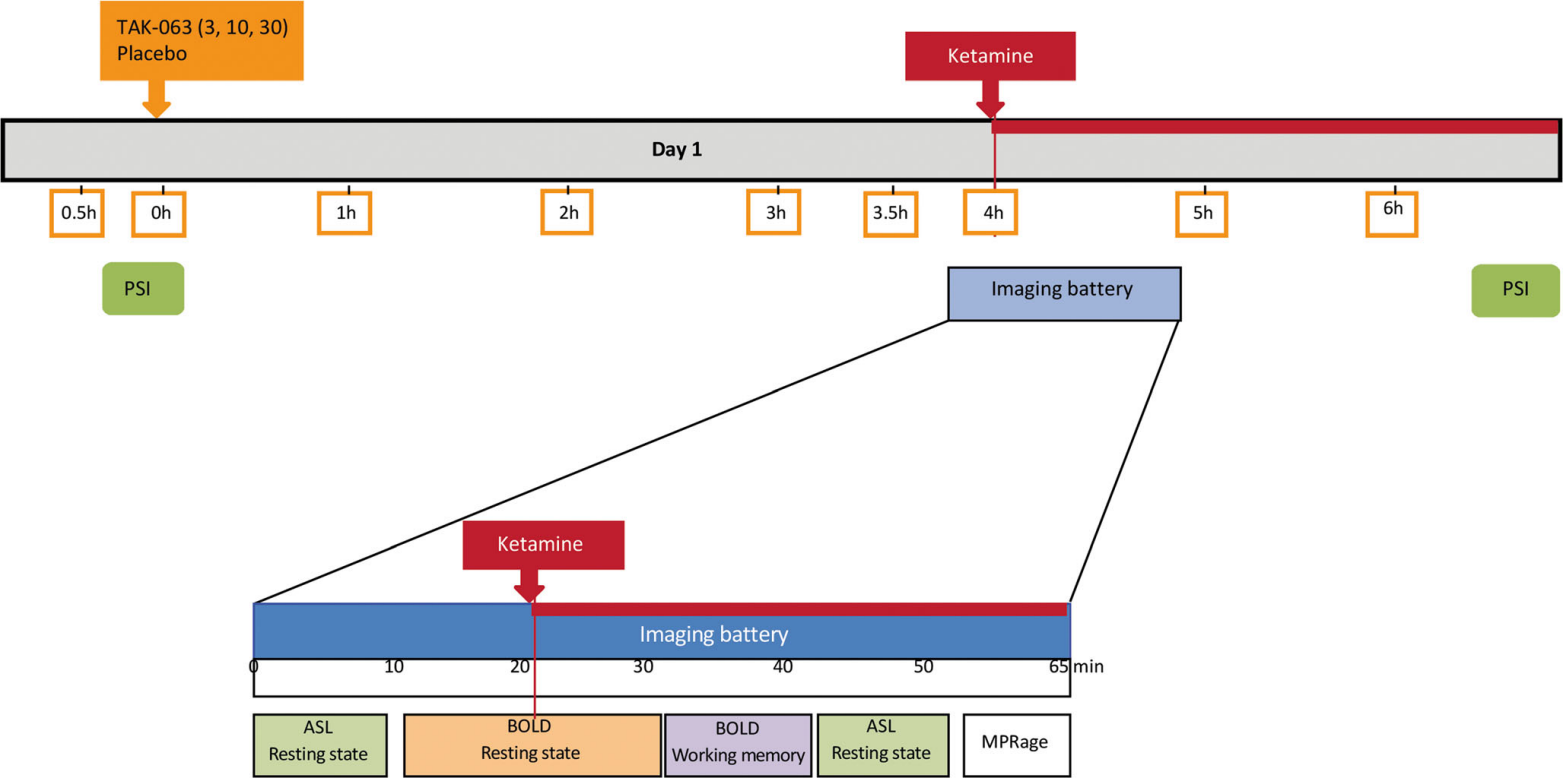
Study design*. ASL*, arterial spin labeling; *BOLD*, blood oxygen level-dependent; *MPRage*, magnetization-prepared rapid acquisition with gradient echo

Subjects checked in on day − 1 of each treatment period during which vital signs, electrocardiogram (ECG), and laboratory safety tests were repeated. On day 1 of each treatment period, subjects received a 3-, 10-, or 30-mg TAK-063 dose or placebo tablets. Participants then relaxed, slept, or watched movies while remaining in the research environment. Ketamine (0.12 mg/kg intravenous bolus over 60 s followed by a continuous infusion of 0.31 mg/kg/h over approximately 150 min) was administered 4 h after TAK-063 or placebo (Fig. 1). This ketamine dose was based on a previous study that achieved steady-state concentrations of 75 ng/mL and provided a robust BOLD signal with minimal psychotomimetic symptoms (De Simoni et al. 2013).

Pharmacodynamic (PD) assessments, such as the neuroimaging battery and memory tasks, were performed before and during ketamine infusion. Safety was continuously assessed during ketamine administration.

### Neuroimaging battery tests

The scanning protocol for the current study was very comprehensive and included multiple MR approaches. This publication reports the BOLD fMRI data for the working memory task, but also resting state fMRI. The magnetic scanning portion of the neuroimaging test battery was initiated 20 min before ketamine dosing. Imaging was performed using a 3 Tesla Siemens Trio scanner. The MR scanning protocol was initiated 20 min before ketamine dosing; prior to scanning, a structural magnetization prepared rapid gradient echo (MPRAGE) scan was collected. As part of the scanning protocol, each subject received 20 min of scanning − 10 min arterial spin labeling (ASL) plus 10 min resting BOLD prior to ketamine infusion. After ketamine infusion, the resting state fMRI BOLD acquisition continued for an additional 10 min. Next, a Spatial Working Memory task-induced fMRI BOLD acquisition was performed for 15 min. Following these BOLD acquisitions, another 10 min ASL scan was acquired and a 10-min T1 relaxometry scan was conducted. The MR scanning sequence ended with a 5-min MPRAGE acquisition.

### Image acquisition

Fifty sequential gradient-echo echoplanar images (EPIs) sensitive to blood oxygenation level-dependent (BOLD) signal were collected in contiguous slices of 6-mm thickness, with 3000-ms repetition time, 40-ms echo time, 20 × 20-cm field of view, a 64 × 64 image matrix, 70° flip angle, and an in-plane resolution of 3 × 3 mm. A working memory challenge based on the expectancy A-X Continuous Performance Test (AX-CPT) paradigm was presented. The AX-CPT task was implemented in Eprime and presents 144 trials in four blocks of 36 trials. Before scanning, participants practiced the task (Barch et al. 2001; MacDonald et al. 2003).

### Image pre-processing

We conducted a number of pre-processing steps to standardize examination of data after acquisition. These steps included (a) using the anatomical image (T1 weighted MPRAGE acquired for each subject at the beginning of the imaging session) to determine the parameters for spatial normalization to the EPI template image supplied with statistical parametric mapping (SPM) using a 12-parameter transformation; (b) registering functional images to correct for subject movement during the experimental paradigm; (c) normalizing functional images to transform images from analyzed subjects into the same standard space; (d) smoothing images (8-mm full width at half maximum [FWHM]) to minimize normalization artifacts and improve signal to noise ratio; (e) applying a high pass filter of 1200 s to the data following spatial smoothing to minimize the influence of very low frequency noise present in fMRI BOLD data without distorting the ketamine response profile.

### Image analyses

The functional imaging analyses for the working memory task was performed using SPM8 (Wellcome Department of Cognitive Neurology, London, UK). Functional images were realigned to correct for motion-related variance components normalized to the standard Montreal Neurological Institute (MNI) EPI template (Talairach and Tournoux 1988; Friston et al. 1995a) and spatially smoothed with an 8-mm FWHM isotropic Gaussian kernel to allow for anatomical variation among subjects. The statistical parametric maps were generated using the general linear model in SPM8 (Friston et al. 1995b). Low-frequency noise was removed with a high-pass filter with a cutoff of 127 s applied to the fMRI time series at each voxel. Data from each subject for each task were analyzed with a fixed box-car function convolved with a model hemodynamic response function. Signal change was acquired from individual ROIs; a total of 22 ROIs were used in the study, including amygdala (left and right), anterior cingulate gyrus (left and right), striatum (caudate and putamen; left and right), dorsolateral prefrontal cortex (BA8 + BA9 + BA46; left and right), posterior cingulate gyrus (left and right), substantia nigra (left and right), ventrolateral prefrontal cortex (BA44 + BA45BA47; left and right), subgenual anterior cingulate cortex (BA25; left and right), thalamus (left and right), paracingulate gyrus (BA32; left and right), and hippocampus (left and right).

### Clinical and behavioral measures

We collected a number of behavioral and side effect measures, including the Psychotomimetic States Inventory (PSI), the Columbia Suicide Severity Rating Scale (C-SSRS), and the CNS Vital Signs Cognitive Battery. These were exploratory measures and are not included in this report.

### Study endpoints

The primary endpoint was ketamine-induced brain activity during the resting state in select regions of the brain including the anterior cingulate cortex, striatum, and ventrolateral prefrontal cortex. Secondary endpoints included pharmacokinetic (PK) parameters of TAK-063 (maximum plasma drug concentration [*C*_max_], average steady-state plasma drug concentration [*C*_av_], time to reach maximum plasma concentration [*t*_max_], area under the plasma concentration-time curve for 24 h [AUC_24_]), and percentage of subjects experiencing at least one treatment-emergent adverse event (AE).

### Pharmacokinetic assessments

Blood samples were collected for the determination of TAK-063 and ketamine concentrations, and ketamine concentrations were used to verify intravenous delivery of ketamine. Blood samples for determination of TAK-063 concentration were collected at pre-dose and at 0.5, 2, 3, 4.5, 6, 8, 10, 12, and 24 h post dose. Blood samples for determination of ketamine concentration were collected at 0.5, 1, 2, and 2.5 h after administration of ketamine. The target ketamine concentration was established at 75 ng/mL. PK samples were not collected from the arm in which ketamine was administered.

### Safety assessments

AEs, clinical laboratory results, ECG, vital signs, and suicide assessments were recorded after administration of TAK-063 and throughout the remainder of the study. Intensity of AEs was classified as mild, moderate, and severe, and each AE was assessed for association with study drug. AEs were assessed throughout the 2-day study visit, at the three treatment visits, at the study exit, and at the follow-up visit. ECG, vital signs, and clinical laboratory results were assessed at visit 1 during screening, day − 1, day 1 of each treatment period, day 2 of period 3 (study exit), the early termination visit, and the follow-up visit.

### Statistical analyses

In this study, the safety set, used for demographic and safety evaluations, included subjects who receive at least one dose of study medication. The PK set included all subjects in the safety set with at least one available drug concentration measurement. The PD set included all subjects in the safety set and with at least one valid PD assessment.

This study was not powered for any comparison, and the number of subjects was considered to be suitable for fulfilling study objectives. All statistical tests were two-tailed at *α* = 0.05 level of significance. For the analysis of the neuroimaging battery test results, effect sizes of > 0.3 were considered potentially meaningful, even without power to detect statistical significance. PK parameters of TAK-063 were generated using WinNonlin Version 6.3. fMRI images were analyzed using SPM8 and Matlab. Percent signal change for resting state data was calculated between pre-ketamine and post-ketamine infusion based on a priori ROIs using SPM’s Anatomy Toolbox (Yurgelun-Todd et al. 2016). Percent signal change for the working memory task was calculated for the task versus baseline in the post-ketamine condition using the same ROI used for resting state (Yurgelun-Todd et al. 2016). An analysis of variance (ANOVA) model was used to perform the analyses for BOLD signal changes (Yurgelun-Todd et al. 2016). Cohen’s *d* effect sizes were calculated as

$$ d=\frac{\hat{\mu_t}-\hat{\mu_p}}{s_p} $$

where *d* is the effect size and $\hat{\mu_t}$ and $\hat{\mu_p}$ are the least-square means for a TAK-063 dose and placebo regimen, respectively, from the ANOVA model. *s*_*p*_ is the pooled standard deviation from both regimens. No adjustments were made for multiple comparisons.

## Results

### Demographics

In total, 27 subjects were enrolled and randomly assigned to treatment schedules as follows: 22 to regimen A (placebo + ketamine), 14 to regimen B (3 mg TAK-063 + ketamine), 15 to regimen C (30 mg TAK-063 + ketamine), and 14 to regimen D (10 mg TAK-063 + ketamine); two subjects received 300 mg of TAK-063 and were not included in the analysis. A total of 20 subjects completed the study (treatment sequences are shown in Online Resource Supplementary Fig S1). All subjects were male, and most were white (88.9%) and non-Hispanic or non-Latino (96.3%) (Online Resource Supplementary Table S2). Mean age, height, weight, and BMI were generally similar across dosing groups (Online Resource Supplementary Table S2).

### Pharmacokinetics

After oral administration, the rate of TAK-063 absorption appeared to be moderate, with a median *t*_max_ value of 3 h (range 0.5–6 h). Dose proportionality analysis for *C*_max_ and AUC of TAK-063 showed a close to dose proportional increase in exposure across the dose range (3–30 mg). After *C*_max_ was reached, plasma concentrations declined in a biphasic manner, with an initial rapid phase followed by a slower terminal phase. AUC_24_ values increased with TAK-063 dose (3 mg: 111.3 ng·h/mL; 10 mg: 549.2 ng·h/mL; 30 mg: 1296.3 ng·h/mL). Average concentration (*C*_av_) values for 3, 10, and 30 mg doses of TAK-063 were 4.63, 22.9, and 53.9 ng/mL, respectively. Overall, the PK profile of TAK-063 was similar to that described in the single-rising dose study, with some differences in TAK-063 M-I PK (Tsai et al. 2016).

Following ketamine administration, mean ketamine concentrations ranged from 69.9 to 83.3 ng/mL at 30 min post dose, irrespective of co-administration of placebo or TAK-063 from 3 to 30 mg. During continuous infusion, plasma concentrations of ketamine reached mean *C*_max_ values ranging from 167.7 to 178.7 ng/mL. *t*_max_ values for ketamine ranged from 2.3 to 2.5 h, and overall *C*_av_ values ranged from 118.6 to 124.6 ng/mL. Inter-subject variability of *C*_max_ and *C*_av_ was generally moderate across doses.

### fMRI BOLD signal

Ketamine administration increased fMRI BOLD resting signal in the placebo group in most regions studied. A decrease in fMRI BOLD was observed in cingulate/BA25. These results are consistent with previous reports (De Simoni et al. 2013).

**Fig. 2:**
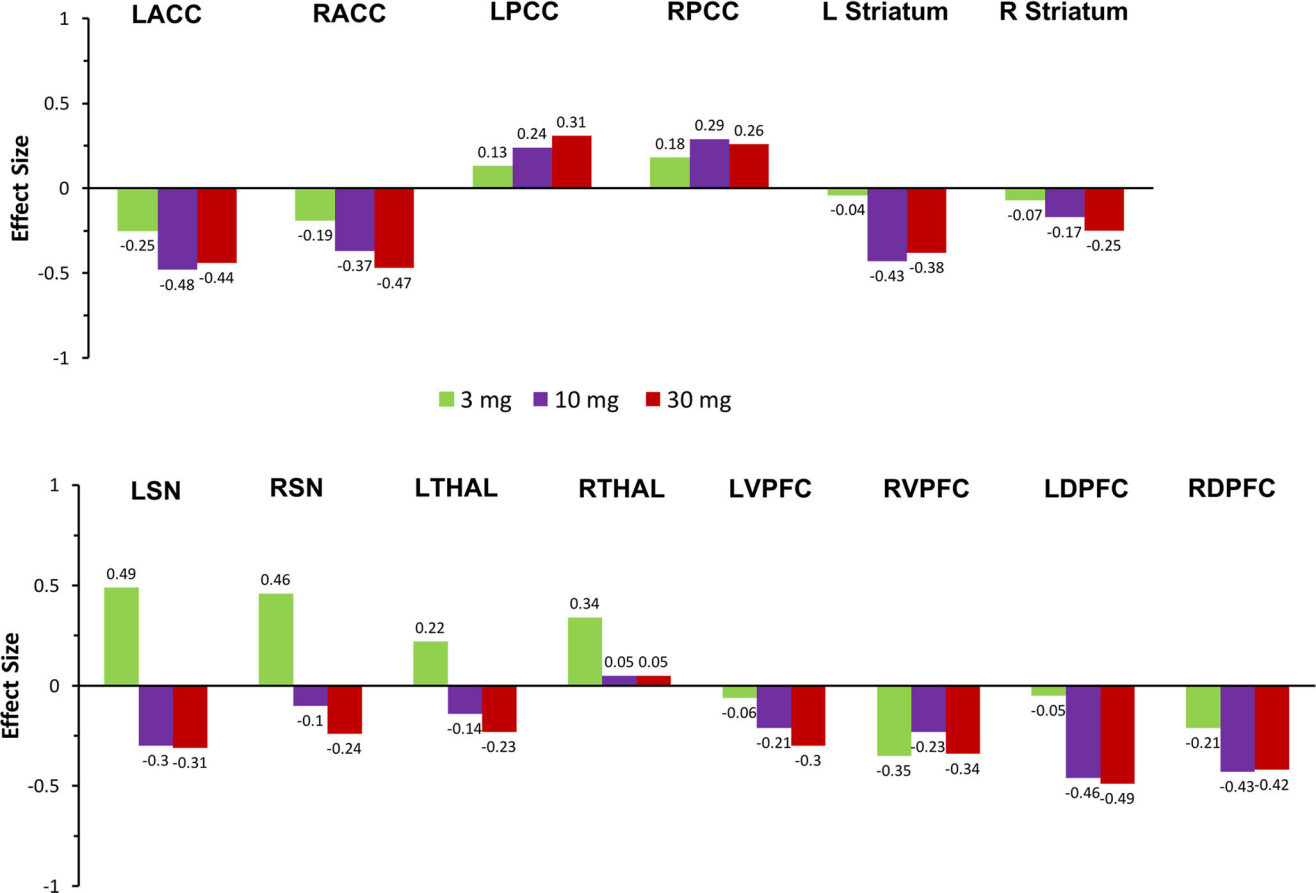
Effects of TAK-063 on resting fMRI BOLD signal before ketamine infusion. Effect sizes were calculated using least squares mean data compared with placebo from the ANOVA model*. fMRI*, functional magnetic resonance imaging; *BOLD*, blood oxygen level-dependent; *LACC*, left anterior cingulate cortex; *LDPFC*, left dorsolateral prefrontal cortex; *LPCC*, left posterior cingulate cortex; *LSN*, left substantia nigra; *L Striatum*, left striatum; *LTHAL*, left thalamus; *LVPFC*, left ventrolateral prefrontal cortex; *RACC*, right anterior cingulate cortex; *RDPFC*, right dorsolateral prefrontal cortex; *RPCC*, right posterior cingulate cortex; *RSN*, right substantia nigra; *R Striatum*, right striatum; *RTHAL*, right thalamus; *RVPFC*, right ventrolateral prefrontal cortex

**Fig. 3:**
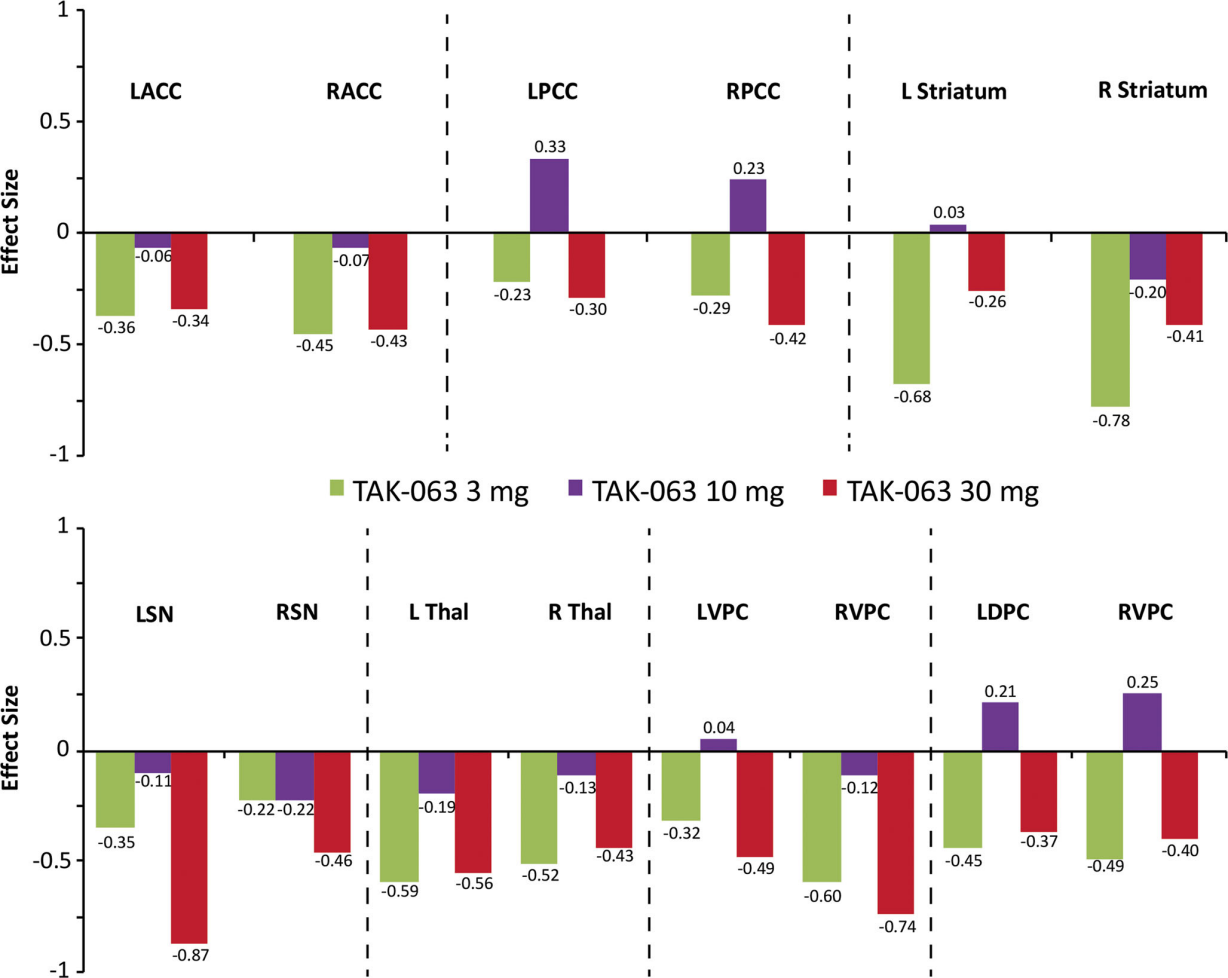
Effects of TAK-063 treatment on ketamine-induced changes in fMRI BOLD signal during resting state. Effect sizes were calculated using least squares mean data as compared to placebo from the ANOVA model*. fMRI*, functional magnetic resonance imaging; *BOLD*, blood oxygen level-dependent; *LACC*, left anterior cingulate cortex; *LDPC*, left dorsolateral prefrontal cortex; *LPCC*, left posterior cingulate cortex; *LSN*, left substantia nigra; *L Striatum*, left striatum; *L Thal*, left thalamus; *LVPC*, left ventrolateral prefrontal cortex; *RACC*, right anterior cingulate cortex; *RPCC*, right posterior cingulate cortex; *RSN*, right substantia nigra; *R Striatum*, right striatum; *R Thal*, right thalamus; *RVPC*, right ventrolateral prefrontal cortex

**Fig. 4:**
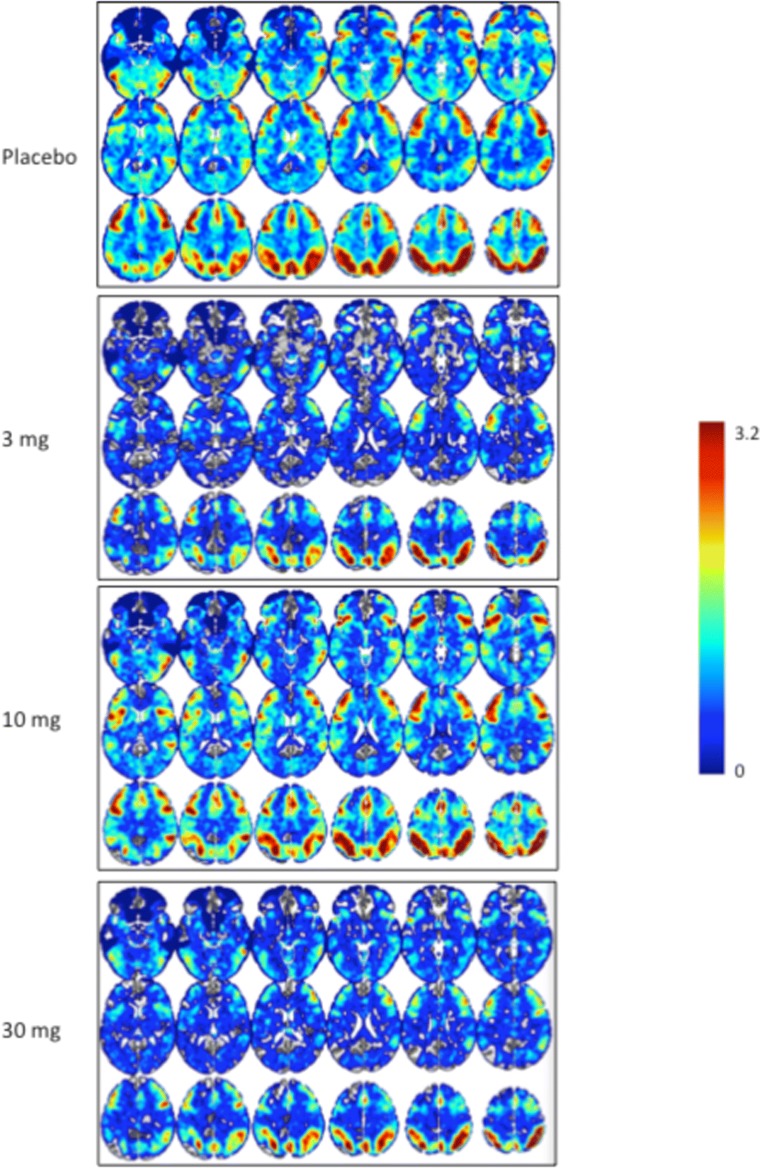
Regional effects of different doses of TAK-063 treatment on ketamine-induced changes in fMRI BOLD signal during resting state*. fMRI*, functional magnetic resonance imaging; *BOLD*, blood oxygen level-dependent

**Fig. 5:**
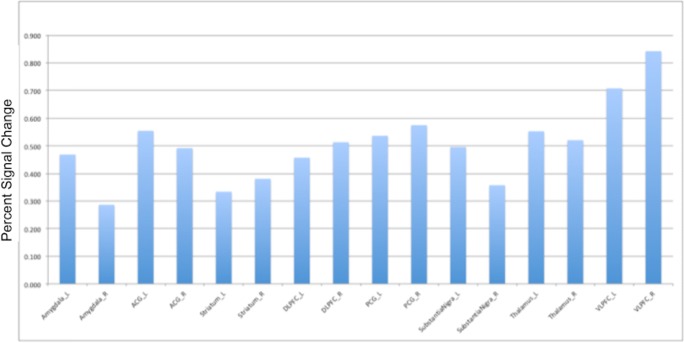
Average ketamine-induced signal change for fMRI BOLD resting state data in the placebo regimen (regimen A)*. fMRI*, functional magnetic resonance imaging; *BOLD*, blood oxygen level-dependent; *ACG*, anterior cingulate gyrus; *DLPFC*, dorsolateral prefrontal cortex; *L*, left; *PCG*, posterior cingulate cortex; *R*, right; *VLPFC*, ventrolateral prefrontal cortex

TAK-063 exerted region-specific effects on fMRI BOLD signal during the resting state before ketamine infusion (Fig. 2). Compared with placebo, TAK-063 increased Cohen’s effect size for resting fMRI BOLD signal across all dose groups at the left and right posterior cingulate cortex and right thalamus. TAK-063 reduced Cohen’s effect size for resting fMRI BOLD signal in all dose groups at the anterior cingulate cortex, striatum, ventrolateral prefrontal cortex, and dorsolateral prefrontal cortex. Following ketamine administration, TAK-063 had a meaningful effect attenuating ketamine-induced changes on resting state fMRI BOLD signals in many key regions examined (Fig. 3). Even though the study was not powered to demonstrate statistical significance, there were noticeable regional fluctuations (Fig. 4), and the following regions showed an absolute effect size greater than 0.3 (threshold established for clinical meaningfulness) and a statistically significant difference (*P* < 0.05) vs placebo: left striatum (3 mg TAK-063; *P* = 0.039; 95% confidence interval [CI] − 0.5955, − 0.0162), right striatum (3-mg TAK-063; *P* = 0.020; 95% CI − 0.5985, − 0.0554), left substantia nigra (30-mg TAK-063; *P* = 0.012; 95% CI − 0.7724, − 0.1031), and right ventrolateral prefrontal cortex (30-mg TAK-063; *P* = 0.031; 95% CI − 0.7912, − 0.0415). However, the effect sizes at the 10-mg TAK-063 dose were generally smaller relative to the effects of other dose groups during assessment of ketamine-induced BOLD changes in the resting state. The average ketamine-induced BOLD signal changes in the placebo regimen (regimen A) are shown in Fig. 5.

**Fig. 6:**
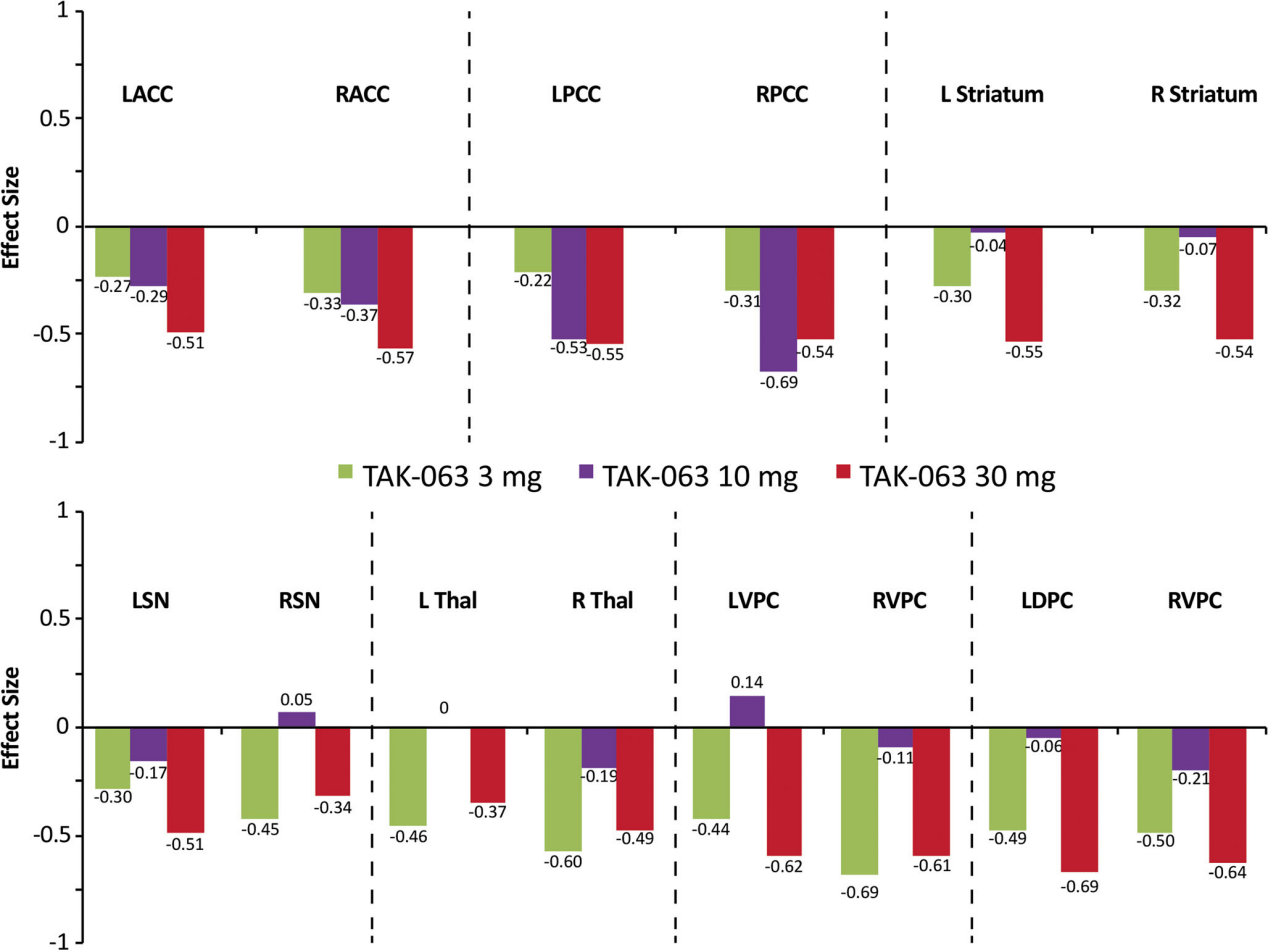
Effects of TAK-063 treatment on ketamine-induced changes in FMRI BOLD signal during the execution of working memory tasks. Effect sizes were calculated using least squares mean data compared with placebo from the ANOVA model*. fMRI*, functional magnetic resonance imaging; *BOLD*, blood oxygen level-dependent; *LACC*, left anterior cingulate cortex; *LDPC*, left dorsolateral prefrontal cortex; *LPCC*, left posterior cingulate cortex; *LSN*, left substantia nigra; *L Striatum*, left striatum; *L Thal*, left thalamus; *LVPC*, left ventrolateral prefrontal cortex; *RACC*, right anterior cingulate cortex; *RPCC*, right posterior cingulate cortex; *RSN*, right substantia nigra; *R Striatum*, right striatum; *R Thal*, right thalamus; *RVPC*, right ventrolateral prefrontal cortex

During the working memory task and across all doses, TAK-063 decreased the Cohen’s effect size for fMRI BOLD signal in many brain regions (Fig. 6). The only exceptions were in the right substantia nigra, left thalamus, and left ventrolateral prefrontal cortex in the 10-mg TAK-063 group, where small non-negative effects (effect size < 0.15) were evident, and the left amygdala, which showed a small non-negative effect in the 30-mg TAK-063 group. The following regions showed an absolute effect size greater than 0.3 and statistically significant difference (*P* < 0.05) vs. placebo: right ventrolateral prefrontal cortex (3-mg; *P* = 0.036; 95% CI − 1.0251, − 0.0369) and left dorsolateral prefrontal cortex (30-mg; *P* = 0.043; 95% CI − 1.0107, − 0.0163). As observed during the resting state, effect sizes for the 10-mg TAK-063 group at many regions were generally smaller compared with other dose groups.

**Fig. 7:**
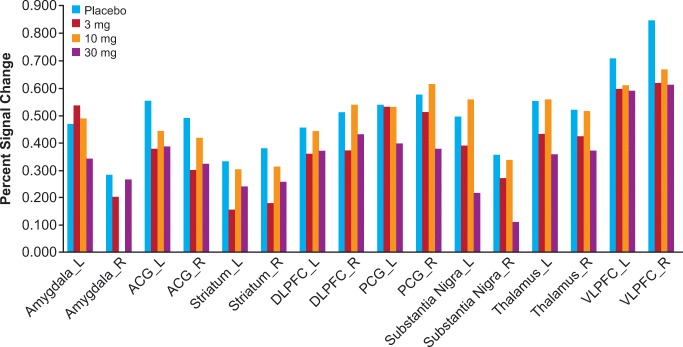
Percent ketamine-induced BOLD signal change for resting state during placebo and TAK-063 regimens in evaluated ROIs*. BOLD*, blood oxygen level-dependent; ROIs, regions of interest; *ACG*, anterior cingulate gyrus; *DLPFC*, dorsolateral prefrontal cortex; *L*, left; *PCG*, posterior cingulate cortex; *R*, right; *VLPFC*, ventrolateral prefrontal cortex

Ketamine increased activation in all ROIs examined (Fig. 7). The attenuation in ketamine-induced BOLD signal changes seen with TAK-063 pre-treatment (vs placebo pretreatment) demonstrated focal patterns of activation similar to what would be expected in healthy control subjects in the absence of ketamine administration (Online Resource Supplementary Fig S2).

Together, these results suggest that TAK-063 ameliorated ketamine-induced changes in fMRI BOLD signal during the resting state and working memory task.

### Safety

A total of 24 subjects (88.9%) experienced AEs that were related to the study drug, and all were mild to moderate in severity. The most frequently reported AEs (≥ 2 subjects) were vomiting (63.0%), nausea (40.7%), somnolence (40.7%), and fatigue (25.9%). No changes from baseline to post dose in vital signs (serum chemistry, hematology, and urinalysis), clinical laboratory tests, and 12-lead ECGs were considered clinically significant. No deaths or serious AEs occurred during the study, and AEs led one participant to discontinue study drug. These results are consistent with the single-rising dose study (Tsai et al. 2016).

Overall, AEs consistent with extrapyramidal symptoms (EPS) were experienced by two subjects in the TAK-063 group (8.0%), and no subjects in the placebo group experienced symptoms of EPS. One subject in the 30-mg TAK-063 group experienced restlessness and another in the 300-mg TAK-063 group experienced muscle tightness and restlessness. One subject receiving 300 mg of TAK-063 discontinued because of EPS. Together, these results suggest that TAK-063 was safe and well tolerated during the study.

## Discussion

The modulation of the BOLD signal by TAK-063 may be related to the augmentation of the direct and indirect striatal output. TAK-063 attenuated ketamine-induced changes in BOLD signal in multiple regions of the brain during the resting state and working memory tasks. This trend was similar to a previous study in which risperidone reduced ketamine-induced BOLD signal in multiple regions of the brain (Doyle et al. 2013). However, the BOLD resting state results from our study do not align with a previous ASL-based study of ketamine-induced changes in brain perfusion in which risperidone increased ketamine-induced perfusion changes (Shcherbinin et al. 2015). Taken together, these results suggest that the attenuation of BOLD changes seen with risperidone, and possibly with TAK-063, is not due to generalized changes in blood flow. The PK of TAK-063 were consistent with those of previous reports (Tsai et al. 2016), and TAK-063 plasma concentrations exhibited biphasic behavior, as has been observed in previous single- and multiple-rising dose studies (Goldsmith et al. 2017; Tsai et al. 2016).

While the study was not powered for any comparison, there was a consistent attenuation of the ketamine-induced signal increases in BOLD signal with TAK-063 administration that is generally consistent with the effects of risperidone on ketamine-induced increases in BOLD (Doyle et al. 2013). Although not definitively known, the attenuation of ketamine-induced increases in BOLD in this translational model of schizophrenia may be suggestive of potential antipsychotic effects of TAK-063. The effects on ketamine-induced changes in BOLD signal were most consistent in the 3- and 30-mg dose groups, especially during the working memory task. Particularly in cortical regions, the effects were generally dose-dependent in the 3- and 30-mg dose groups. In general, the magnitude of change in the 10-mg group was less, although the 10-mg TAK-063 dose results were more consistent in the cortical regions of the brain; the reasons for these differences are not known.

TAK-063 was well tolerated at all doses of the study. No serious AEs were experienced by subjects receiving TAK-063, and most AEs were of mild to moderate severity. Only two subjects experienced symptoms of EPS, and one of the two subjects received a relatively high TAK-063 dose (300 mg) that was later changed to 10 mg (according to protocol amendment 3). The safety profile of TAK-063 closely resembled that of the single-rising dose study in which only one subject experienced EPS-like symptoms (Tsai et al. 2016).

PDE-10 is selectively expressed on the medium spiny neurons of the striatum. This enzyme inhibits the dephosphorylation of G-protein receptors, which are coupled to the inhibition of cAMP and cGMP, and therefore act in general like D2 dopamine antagonists (Nishi et al. 2008; Sano et al. 2008; Xie et al. 2006; Gresack et al. 2014). To date, PDE10A inhibitors have not shown clinical efficacy in the treatment of schizophrenia, and the predictability of this translational model of schizophrenia has not been definitively established. TAK-063 is a potent, highly selective, and orally active PDE10A inhibitor that produces a balanced activation of downstream cAMP and CGMP signals in both direct and indirect pathways medium spiny neurons—balanced activation of these pathways may be critical for an antipsychotic-like effect (Suzuki and Kimura 2018; Suzuki et al. 2016). In preclinical models, TAK-063 has shown effects that are consistent with potential antipsychotic efficacy, and has also attenuated the increases in BOLD in anesthetized rats; additionally, in clinical studies of individuals with schizophrenia, potential antipsychotic-like effects were observed. In a 6-week phase 2 study, the difference between the 20-mg dose of TAK-063 and placebo did not meet its primary endpoint (change from baseline in Positive and Negative Syndrome Scale [PANSS] score at week 6). However, statistically significant effects were observed for several secondary endpoints, and the overall results were suggestive of antipsychotic efficacy. Unfortunately, the interpretation of this study was confounded by a relatively large change from baseline in PANSS score in the placebo group, the lack of dose-ranging comparisons, and an active reference (Macek et al. 2017). Further clinical studies are required to assess the potential antipsychotic effects of TAK-063.

Limitations of this study include the small sample size and the significant dropout rate (20 out of 27 subjects completed the study). All subjects were male (homogeneous sample), which may not allow to generalize findings to females. The exposure to ketamine was longer than that used in other studies (4 h as opposed to 40 min), making comparisons across studies difficult. Given the observed side effects with the 300-mg dose of TAK-063, we were unable to complete the dose-response curve that was initially planned (3–300 mg). Additionally, no adjustments for multiplicity were made for statistical analyses.

Additional analyses are required to examine the possible contributions of changes in blood flow to the observations. ASL was measured in this study, and further analyses of these data are the next logical step for assessing the impact of the findings. These results may help to further understand the PD effects of TAK-063 and other PDE10A inhibitors.

## Electronic supplementary material

Supplementary Fig S1. CONSORT diagram for the EEG study of TAK-063 in healthy adults (PNG 532 kb)

High Resolution Image (TIF 504 kb)

Supplementary Fig S2. Effects of TAK-063 on ketamine-induced BOLD changes during the working memory task. The images display the within-group statistical map generated for the working memory task, showing areas of statistically significant activation after pretreatment with 10 mg TAK-063 (*n* = 14). Minimal cluster size was set to 20 voxels and significance was reported on *P* < 0.001 level*. BOLD*, blood oxygen level-dependent (PDF 199 kb)

ESM 1 (DOC 52 kb)
