# Supplementary figures and images for: A randomized, placebo-controlled, phase 1 study to evaluate the effects of TAK-063 on ketamine-induced changes in fMRI BOLD signal in healthy subjects
Source: Psychopharmacology (Berl). 2019 Nov 26;237(2):317–28. doi: 10.1007/s00213-019-05366-1 (PMC7018803; doi:10.1007/s00213-019-05366-1)

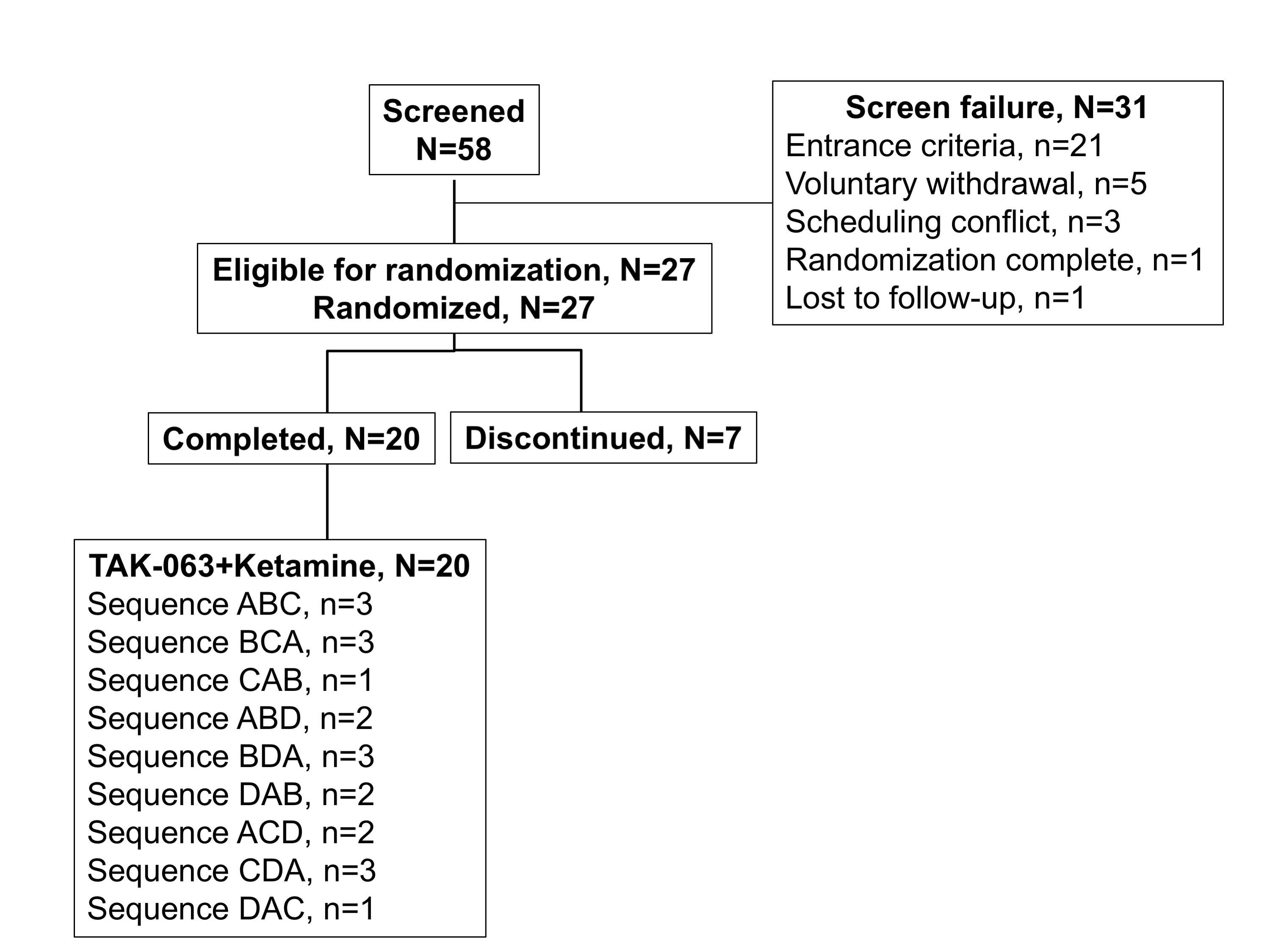

Supplement: Supplementary file 1 — CONSORT diagram for the EEG study of TAK-063 in healthy adults (PNG 532 kb) [file 213_2019_5366_Fig8_ESM.png]

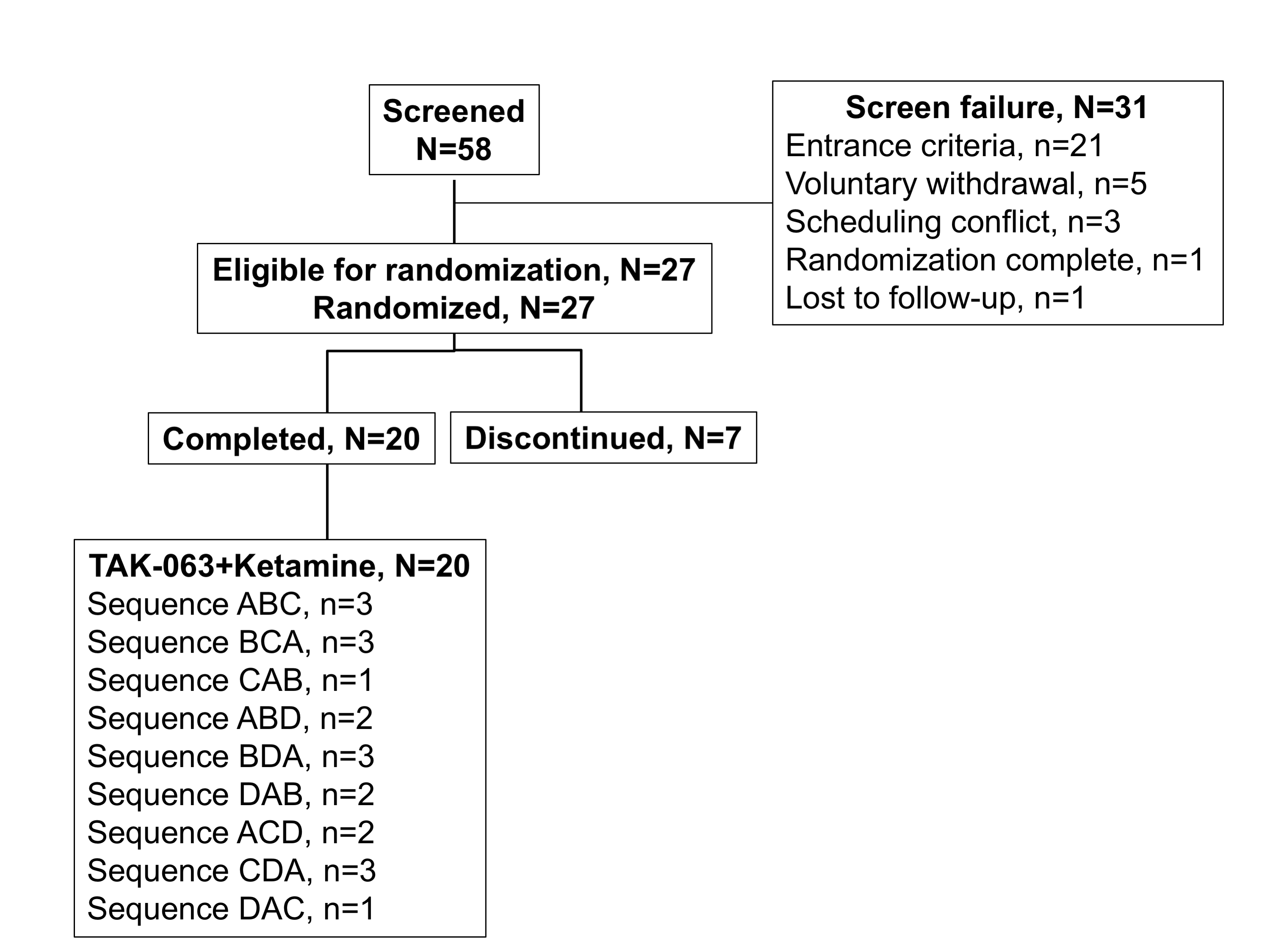

Supplement: Supplementary file 2 — High Resolution Image (TIF 504 kb) [file 213_2019_5366_MOESM1_ESM.tif]

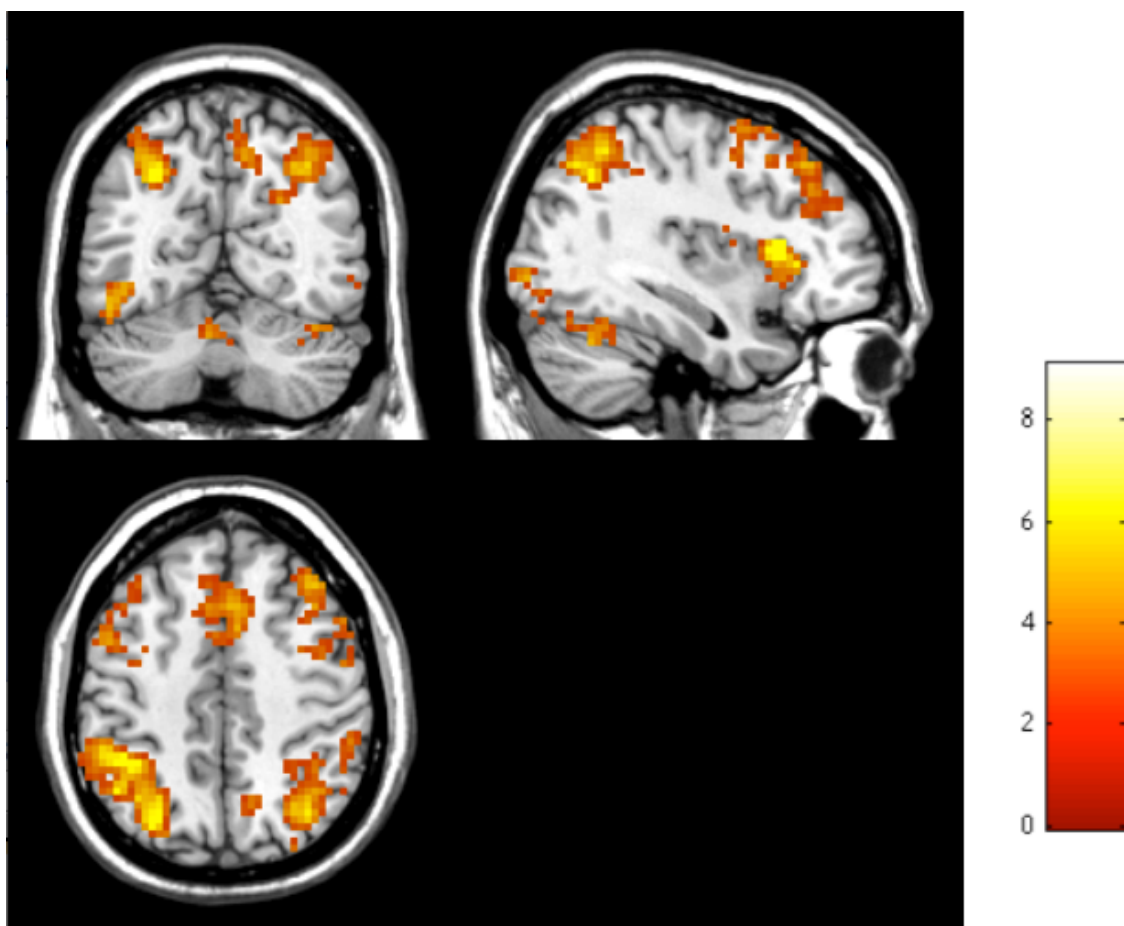

Supplement: Supplementary file 3 — Effects of TAK-063 on ketamine-induced BOLD changes during the working memory task. The images display the within-group statistical map generated for the working memory task, showing areas of statistically significant activation after pretreatment with 10 mg TAK-063 (n = 14). Minimal cluster size was set to 20 voxels and significance was reported on P < 0.001 level. BOLD, blood oxygen level-dependent (PDF 199 kb) [file 213_2019_5366_MOESM2_ESM.pdf]
