# Supplementary material for: A randomized, placebo-controlled, phase 1 study to evaluate the effects of TAK-063 on ketamine-induced changes in fMRI BOLD signal in healthy subjects
Source: Psychopharmacology (Berl). 2019 Nov 26;237(2):317–28. doi: 10.1007/s00213-019-05366-1 (PMC7018803; doi:10.1007/s00213-019-05366-1)
Supplement: Supplementary file 4 — (DOC 52 kb) [file 213_2019_5366_MOESM3_ESM.doc]

Supplementary Table S1. Treatment Sequences

| **Sequence** | **Period 1** | **Period 2** | **Period 3** |
| --- | --- | --- | --- |
| 1 | A | B | C |
| 2 | B | C | A |
| 3 | C | A | B |
| 4 | A | B | D |
| 5 | B | D | A |
| 6 | D | A | B |
| 7 | A | C | D |
| 8 | C | D | A |
| 9 | D | A | C |

A: Placebo + Ketamine; B: 3-mg TAK-063 + Ketamine; C: 30-mg TAK-063 + Ketamine; D: 300-mg TAK-063 + Ketamine.

Supplementary Table S2. Demographic and Baseline Characteristics

|  | **ABC** | **BCA** | **CAB** | **ABD** | **BDA** | **DAB** | **ACD** | **CDA** | **DAC** | **Total** |
| --- | --- | --- | --- | --- | --- | --- | --- | --- | --- | --- |
|  | *n*=3 | *n*=3 | *n*=3 | *n*=3 | *n*=3 | *n*=3 | *n*=3 | *n*=3 | *n*=3 | *N*=27 |
| **Characteristic** | *n* (%) | *n* (%) | *n* (%) | *n* (%) | *n* (%) | *n* (%) | *n* (%) | *n* (%) | *n* (%) | *n* (%) |
| Age, years, mean (SD) | 27.7 (3.21) | 28.7 (7.64) | 23.7 (6.66) | 32.3 (5.13) | 23.0 (5.20) | 22.3 (3.51) | 27.0 (7.94) | 24.7 (2.08) | 23.7 (5.69) | 25.9 (5.60) |
| Sex, (n %) |  |  |  |  |  |  |  |  |  |  |
| Male | 3  (100) | 3  (100) | 3  (100) | 3  (100) | 3  (100) | 3  (100) | 3  (100) | 3  (100) | 3  (100) | 27  (100) |
| Ethnicity, *n* (%) |  |  |  |  |  |  |  |  |  |  |
| Hispanic or Latino | 0 | 0 | 0 | 0 | 0 | 0 | 1  (33.3) | 0 | 0 | 1  (3.7) |
| Non-Hispanic  or Latino | 3  (100) | 3  (100) | 3  (100) | 3  (100) | 3  (100) | 3  (100) | 2  (66.7) | 3  (100) | 3  (100) | 26 (96.3) |
| Height, mean (SD), cm | 179.0 (2.65) | 182.0 (4.58) | 177.0 (8.19) | 187.7 (3.79) | 179.7 (10.79) | 182.3 (3.06) | 183.3 (8.14) | 183.3 (6.51) | 182. 7 (4.51) | 181.9 (6.06) |
| Weight, mean (SD), kg | 83.2 (11.98) | 93.8 (4.78) | 79.9 (16.91) | 82.3 (12.64) | 87.5 (13.96) | 77. 3 (8.10) | 81.1 (5.82) | 86.4 (4.93) | 79.1 (11.71) | 83.4 (10.3) |
| BMI, mean (SD), kg/m^2^ | 26.1 (4.43) | 28.4 (2.56) | 25.3 (3.19) | 23.4 (3.84) | 27.0 (2.11) | 23.3 (2.61) | 24.2 (2.42) | 25.7 (1.01) | 23.7 (3.27) | 25.2 (3.00) |
| Smoking, n (%) |  |  |  |  |  |  |  |  |  |  |
| Never smoked | 3  (100) | 2  (66.7) | 3  (100) | 2  (66.7) | 3  (100) | 3  (100) | 2  (66.7) | 3  (100) | 2  (66.7) | 23 (85.2) |
| Ex-smoker | 0 | 1  (33.3) | 0 | 1  (33.3) | 0 | 0 | 1  (33.3) | 0 | 1  (33.3) | 4  (14.8) |

A: Placebo + Ketamine; B: 3 mg TAK-063 + Ketamine; C: 30 mg TAK-063 + Ketamine; D: 10 mg TAK-063 + Ketamine.

BMI, body mass index; SD, standard deviation.
